# Trichinæ in a Cat

**Published:** 1881-03

**Authors:** 


### TRICHINÆ IN A CAT.

Living trichinæ, says the *Pathologist*, have been recently found, at the pathological laboratory of the Long Island College Hospital, in the muscles of a cat which had "died from natural causes," and been brought to the laboratory for dissection. The animal was emaciated and icteric. Placed on a slide, in a neutral solution, the trichinæ kept up a spiral, vermicular movement for several hours.

